# Impact of bismuth-doping on enhanced radiative recombination in lead-free double-perovskite nanocrystals†

**DOI:** 10.1039/d2na00238h

**Published:** 2022-06-24

**Authors:** Xiaoyu Huang, Yoshitaka Matsushita, Hong-Tao Sun, Naoto Shirahata

**Affiliations:** International Center for Materials Nanoarchitectonics (MANA), National Institute for Materials Science (NIMS) Ibaraki 305-0044 Japan SHIRAHATA.Naoto@nims.go.jp; Graduate School of Chemical Sciences and Engineering, Hokkaido University Sapporo 060-0814 Japan; Research Network and Facility Services Division, National Institute for Materials Science (NIMS) Ibaraki 305-0047 Japan; Department of Physics, Chuo University Tokyo 112-8551 Japan

## Abstract

Lead-free double-perovskite nanocrystals (NCs) have received considerable attention as promising candidates for environmentally friendly optical applications. Furthermore, double-perovskite nanostructures are known to be physically stable compared to most other inorganic halide perovskites, with a generic chemical formula of ABX_3_ (*e.g.*, A = Cs^+^; B = Sn^2+^ or Ge^2+^; X = Cl^−^, Br^−^, I^−^, or their combination). However, relevant experimental studies on the photophysical properties are still insufficient for Pb-free double-perovskite NCs. Herein, we synthesized Cs_2_Ag_0.65_Na_0.35_InCl_6_ NCs doped with bismuth (Bi^3+^) ions and investigated their photophysical properties to reveal the role of the dopant on the enhanced photoemission properties. Specifically, it was found that the photoluminescence quantum yield (PLQY) increased up to 33.2% by 2% Bi-doping. The optical bandgap of the NCs decreased from 3.47 eV to 3.41 eV as the amount of the dopant increased from 2% to 15%. To find out the effect of Bi-doping, the temperature-dependent PL properties of the undoped and doped NCs were investigated by utilizing steady-state and time-resolved PL spectroscopy. With increasing the temperature from 20 K to 300 K, the PL intensities of the doped NCs decreased slower than the undoped ones. The correlated average PL lifetimes of both the bismuth-doped and undoped NCs decreased with increasing the temperature. The experimental results revealed that all the NC samples showed thermal quenching with the temperature increasing, and the PL quenching was suppressed in bismuth-doped NCs.

## Abstract 2

We synthesized the Pb-free perovskite NCs doped with bismuth ions and investigated their photophysical properties to reveal the role of the dopant on the enhanced photoemission properties.

## Graphical abstract

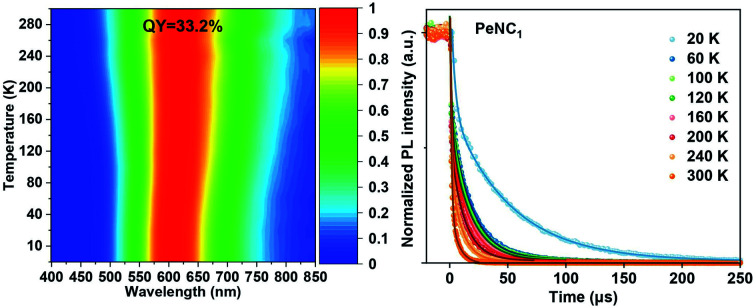

## 1. Introduction

Lead halide perovskite (LHP) nanocrystals (NCs) APbX_3_ (A = Cs^+^, CH_3_NH_3_^+^ or NH_2_HCNH_2_^+^, X = Cl^−^, Br^−^ or I^−^) have become up-and-coming materials in various optoelectronic applications, including light-emitting devices,^1–6^ solar cells,^7–12^ photodetectors,^13–17^ photocatalysis,^18–20^ and lasers,^21–23^ which is attributed to their distinguished optical and electrical properties, such as high defect tolerance,^24,25^ high photoluminescence (PL) quantum yield (QY),^26–28^ tunable bandgap,^29,30^ high color purity,^31,32^ high carrier mobility, large diffusion length,^33^ and high absorption coefficient,^34^ as well as solution processability.^35,36^ Despite the many above-mentioned advantages, the toxicity and instability of lead halide perovskite NCs are still a concern and represent a non-negligible hindrance toward their practical applications.^37,38^

To surmount these obstacles, stable and environmentally friendly perovskite compositions are urgently desired. To replace Pb^2+^, the obvious approach is to use Sn^2+^ and Ge^2+^ because of their similar electronic properties.^39,40^ However, Sn^2+^- and Ge^2+^-based halide perovskites are quite unstable and are easily oxidized to Sn^4+^ and Ge^4+^. Additionally, trivalent cations Bi^3+^ and Sb^3+^ halide perovskites have also been studied,^41^ but compounds with the chemical formula A_3_B_2_X_9_ have shown unfavorable properties, such as a large bandgap and low defect tolerance.^42^ In particular, replacing two Pb^2+^ ions with a combination of monovalent and trivalent cation could be one solution for forming three-dimensional (3D) perovskite structures, whose chemical formula is A_2_B(i)B(iii)X_6_, which is called the double-perovskite structure. For instance, Cs_2_AgBiCl_6_ and Cs_2_AgInCl_6_ compounds have shown good photophysical characteristics after effective alloying or doping engineering.^43,44^

**Scheme 1:**
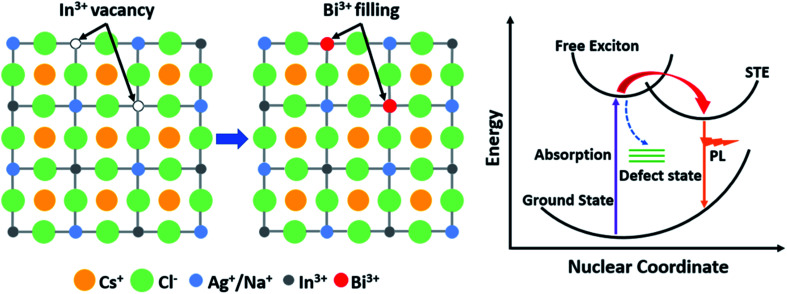
Schematic diagram of the Bi-doping effect and proposed self-trapped emission.

Among the reported lead-free double-perovskite materials, it has been reported recently that the bulk crystals of alloyed Cs_2_Ag_*x*_Na_1−*x*_InCl_6_:Bi double perovskites show a high PLQY of 86 ± 5% with self-trapped exciton (STE) emitting warm white light.^45^ STE is normally formed by fast self-trapped excitons in materials that possess a soft lattice and strong exciton-coupling characteristics, such as halide crystals^46^ and organic molecular crystals,^47^ wherein the excitons relax back to the ground state by emitting photons with a broad band and large Stokes shift after being trapped; therefore the emission energy of an STE is smaller than the bandgap (Scheme 1).^48^ In 2022, Bi^3+^-doped Cs_2_Ag_0.4_Na_0.6_InCl_6_ microcrystals (∼10 μm) with a remarkable high PLQY of 97.33% were achieved by Peng *et al.* through a precipitation approach.^43^ Since then, double-perovskite NCs have also become a potential material for single-emitter layers applied in optical applications.^49^

Colloidal perovskite NCs terminated with surfactant ligands for high dispersibility in non-polar solvents are promising for optoelectronic applications, due to their convenient deposition on substrates based on solution processes.^50^ Thereafter, for further enhancing the practicality in applications, there are efforts being directed toward the colloidal synthesis of double-perovskite NCs.^51–53^ More recently, Hu *et al.* synthesized white light-emitting Cs_2_Ag_0.17_Na_0.83_In_0.88_Bi_0.12_Cl_6_ NCs with a PLQY of 64% by elemental ratio optimization and ligand passivation.^54^ In the double-perovskite structure system, it has been reported that Bi-doping is responsible for the increased PLQY.^51,55,56^ For the underlying mechanism of improving the PLQY through doping bismuth, there are several reported viewpoints: one is defect passivation as proved by the comparison of PL lifetimes, and another is that exciton localization is promoted after Bi-doping, which was concluded from theoretical simulations.^45^ Theory predicts the radiative recombination of photogenerated carriers in the Bi–Ag centers providing that the Bi cations are present in only a small amount.^53^ However, understanding and designing the experimentally fundamental photophysical properties of Bi-doped double-perovskite NCs, which could be favorable for understanding the doping effect, are still challenging themes.

A temperature-dependent PL study was established to discuss the nonradiative relaxation processes and electron–phonon interactions in the quantum dots of semiconductors.^57–59^ In the present study, we used the temperature-dependent PL approach to reveal the possible excitation and emission mechanism arising from the doping of Bi^3+^ ions in Cs_2_Ag_0.65_Na_0.35_InCl_6_ NCs. Changes in the PL intensity and linewidth as a function of temperature were measured to gain insights into the carrier relaxation and electron–phonon coupling processes. Additionally, the temperature-dependent time-resolved PL spectra of the NCs were measured. Furthermore, the structural characterizations of the NCs at different temperatures were analyzed by the low-temperature X-ray diffraction (LT-XRD) technique to clarify the crystal-phase condition with varying the temperature.

## 2. Experimental

### 2.1 Reagents and chemicals

All the reagents were used without further purification. Cesium acetate (CsOAc, 99.99%), silver acetate (AgOAc, 99%), sodium acetate (NaOAc, 99.99%), indium(iii) acetate (In(OAc)_3_, 99.99%), bismuth(iii) acetate (Bi(OAc)_3_, 99.99%), chlorotrimethylsilane (TMSCl, 98%), oleic acid (OA, 90%), and 1-octadecane (ODE, 95%) were purchased from Sigma-Aldrich. Oleylamine (OAm, 80–90%) was purchased from Kanto Kagaku. Toluene (99.5%), hexane (96%), and ethyl acetate (99.5%) were purchased from Wako Chemicals.

### 2.2 Preparation of Cs_2_Ag_0.65_Na_0.35_In_1−*x*_Bi_*x*_Cl_6_ NCs

The NCs were fabricated following a modified version of the hot-injection method reported by Han and co-workers.^52^ In a typical synthesis, 1.3 mmol CsOAc, 0.36 mmol AgOAc, 0.54 mmol NaOAc, 1.98 mmol In(OAc)_3_, and 0.02 mmol Bi(OAc)_3_ were loaded in a 50 mL three-neck flask containing 5.6 mL OA, 1.4 mL OLA, and 20 mL ODE, after degassing for 1 h at 110 °C, and then heated to 165 °C in 12 min under a N_2_ flow. Next, 0.8 mL TMSCl was injected quickly, with the temperature increasing for 2 min longer, and then the production was rapidly cooled down to 25 °C by placing in an ice-water bath. The resultant NCs were centrifuged at 9000 rpm for 20 min and washed with toluene to remove any unreacted precursors. Then, the collected precipitate was further dispersed in hexane, and thereafter the resultant solution was centrifuged at 6000 rpm for 15 min and the supernatant was collected. The same volume of ethyl acetate was added for the final centrifugation, and the final precipitate was collected and vacuum-dried for further characterization.

### 2.3 Characterization

The powder samples with different Bi-doping amounts were analyzed using a JASCO V-650 UV-visible spectrometer. High-resolution transmission electron microscopy (HR-TEM) characterization was performed using a JEM-2100F2 instrument (JEOL) at 200 kV acceleration voltage. Scanning transmission electron microscopy energy dispersive spectroscopy (STEM-EDS) was utilized for the element analysis. Samples for the TEM analysis were prepared by drop-casting the 0.1 mg mL^−1^ hexane solution of NCs onto carbon-coated copper grids. PLQYs were measured at room temperature using the absolute PLQY spectrometer C11347-11 from Hamamatsu Photonics Co. Ltd with a 150 W xenon light source coupled to a monochromator for wavelength discrimination, an integrating sphere as a sample chamber, and a multichannel spectroscope for signal detection. The powder forms of the specimens of NCs were used for the characterization of the PLQY. The PL properties were measured with the powder samples pressed and coated onto the interlayer between two 1 cm × 1 cm quartz glasses. Measurements were conducted using a modular double-grating Czerny–Turner monochromator and an iHR 320 emission monochromator (1200 lines per mm of gratings) coupled to a photodetector on a NanoLog Horiba Jobin Yvon spectrofluorometer with a 450 E Xe arc lamp. The value of photon power for the excitation estimated using a power meter (PD 300, Ophir Optronics Solutions Ltd and NOVA II display) was 0.014 mW cm^−2^. To measure the temperature-dependent PL spectra, the samples were placed inside a cryostat holder connected to a Gifford–McMahon cooler and controlled by a Mercury iTC temperature controller. The temperature was tuned from 3 K to 300 K with 20 K per step. The LT-XRD characterization in the temperature range between 5 K and 300 K was carried out using a Rigaku SmartLab system (9 kW, Cu Kα_1_) equipped with a cryostat attachment, where the sample was tightly attached using Apiezon-N grease on a copper sample holder.

## 3. Results and discussion

**Fig. 1:**
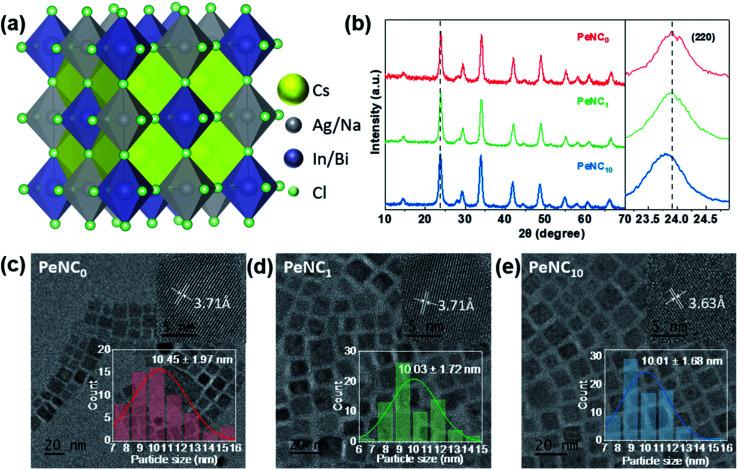
Crystal structure of Cs_2_Ag_1−*x*_Na_*x*_In_1−*y*_Bi_*y*_Cl_6_ double perovskite. Yellow, silver, purple, and green spheres represent Cs, Ag/Na, In/Bi, and Cl atoms, respectively. (b) X-ray diffraction (XRD) patterns measured at room temperature of PeNC_*x*_ (Cs_2_Ag_0.65_Na_0.35_In_1−*x*%_Bi_*x*%_Cl_6_, *x* = 0, 1, 10) and the enlarged XRD patterns of the samples between 23° and 25°. Transmission electron microscopy (TEM) images of (c) PeNC_0_, (d) PeNC_1_, and (e) PeNC_10_. The scale bar is 20 nm. The inset in the top-right corner is the high-resolution TEM image, whose scale bar is 5 nm. The inset in the bottom right corner is the size-distribution histogram.

Bi-doped and undoped Cs_2_Ag_0.65_Na_0.35_InCl_6_ NCs were synthesized by the modified hot-injection method.^52^ Here, PeNC_0_, PeNC_1_, and PeNC_10_ represent Cs_2_Ag_0.65_Na_0.35_InCl_6_ double-perovskite NCs with 0%, 1%, and 10% Bi precursor concentration, respectively. The STEM-EDS analysis revealed that the final products of PeNC_1_ and PeNC_10_ had 2% and 15% Bi. It should be noted that all the composition values stand for the actual value for the results that we obtained from STEM-EDS as listed in Table S1.† The atom ratios of Cs/In and Cl/In showed the ranges of 2.32–2.48 and 4.86–5.61, respectively. This deviation with the feeding ratio was reasonable compared with other double-perovskite nanocrystals.^60,61^ On the one hand, the large ratio of Cs/In might be caused by the indium vacancies in this structure, and this ratio decreased with the increasing bismuth amount. Meanwhile, the elemental ratio deviation was also found in Cs_2_AgBiCl_6_, whereas the ratios were 2.25 and 20 for Cs/Ag and Cl/Ag, respectively.^60^ However, the cause of the large ratio on Cl/In has not been clearly clarified yet, and needs to be investigated in future work. HR-TEM images of Cs_2_Ag_0.65_Na_0.35_InCl_6_ NCs showed that the crystals had a cubic structure. As shown in Fig. 1c–e, the average sizes of PeNC_0_, PeNC_1_, and PeNC_10_ were 10.45 ± 1.97, 10.03 ± 1.72, and 10.01 ± 1.68 nm, respectively. Their sizes were quite close to each other. Based on that, the particle size difference was not considered in the subsequent discussion of the optical properties. Additionally, the corresponding measured lattice distances were 3.71, 3.71, and 3.63 Å, which were in accord with the (220) planes in Cs_2_Ag_0.65_Na_0.35_InCl_6_ NCs, as shown by XRD in the following part. The XRD patterns of Cs_2_Ag_0.65_Na_0.35_InCl_6_ NCs matched well with the cubic double-perovskite structure (Cs_2_AgInCl_6_ ICSD code 1546186, Cs_2_NaInCl_6_ ICSD code 4003575) in all three samples, and no impurity phases were observed (see Fig. 1b), which demonstrated the good phase stability of the Cs_2_Ag_0.65_Na_0.35_InCl_6_ double-perovskite. Comparing the XRD pattern of PeNC_1_ with that of PeNC_10_, we could see a small shift of the 220 peak from 23.9° to 23.8° with increasing the bismuth amount from 2% to 15%, which was possibility due to the lattice expansion resulting from the substitution of Bi^3+^ ions (117 pm) for In^3+^ ions (92 pm) in the cubic lattice.^62^ Cs_2_Ag_0.65_Na_0.35_InCl_6_ NCs with a higher concentration of Bi-dopant were also synthesized, and the lattice parameters of NCs as a function of the Bi-dopant concentration (see Fig. S1†) were investigated, which showed that when the Bi amount increased, the crystal structure of the NCs obeyed Vegard's law,^63^ and there was no impurity phase or phase separation.

**Fig. 2:**
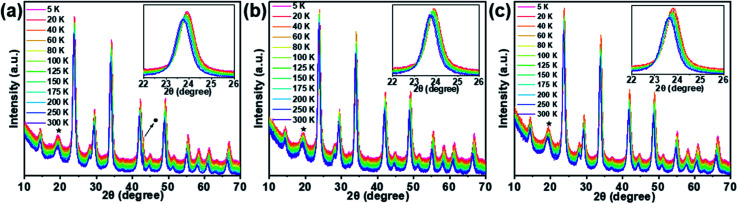
LT-XRD patterns of (a) PeNC_0_, (b) PeNC_1_, and (c) PeNC_10_ in the temperature range from 5–300 K. Peaks indicated as ★ and ● are from the X-ray window materials of the cryostat and from the Cu sample holder, respectively.

It is common that structural phase transition happens in lead halide perovskite NCs under low temperature, which is caused by size effects or doping effects.^64–66^ A phase transition from tetragonal- to cubic structures has been observed at 122 K for Cs_2_AgBiBr_6_ which is one of the double-perovskite crystals.^67^ Such a temperature-dependent phase transition influences adversely the device performance in some cases. To investigate the structural stability of the Cs_2_Ag_0.65_Na_0.35_InCl_6_ NCs when the temperature varies, LT-XRD measurements were performed. For the LT-XRD measurements between 5 K and 300 K, the PeNC_0_, PeNC_1_, and PeNC_10_ samples were selected (see Fig. 2a–c). All the LT-XRD patterns of the doped samples were found to be similar to that of the undoped sample. The absence of additional peaks appeared over the whole temperature range for the doped samples. This indicates that the phase stayed in the cubic phase, implying a good crystallinity and purity. At 5 K, when the Bi amount increased to 2%, the diffraction lines of the PeNC_1_ showed a relative shift compared to that of the PeNC_0_, with the 15% Bi-doped PeNC_10_ showing a 0.22° smaller shift than PeNC_0_, which was consistent with the characteristics shown at room temperature (see Fig. S2†). On the temperature dependence, all the XRD peaks shifted toward a smaller diffraction angle with increasing the temperature, possibly due to a lattice expansion. The experimental data of the lattice constant were fitted by a second-order polynomial, as shown in Fig. S3.† The linear coefficient of thermal expansions for PeNC_0_, PeNC_1_, and PeNC_10_ was found to be 1.71 × 10^−5^/K, 1.27 × 10^−5^/K, and 1.52 × 10^−5^/K, respectively. The results from these calculations indicated that the doped sample changed slowly with temperature, which would permit high thermal stability for their target applications.

**Fig. 3:**
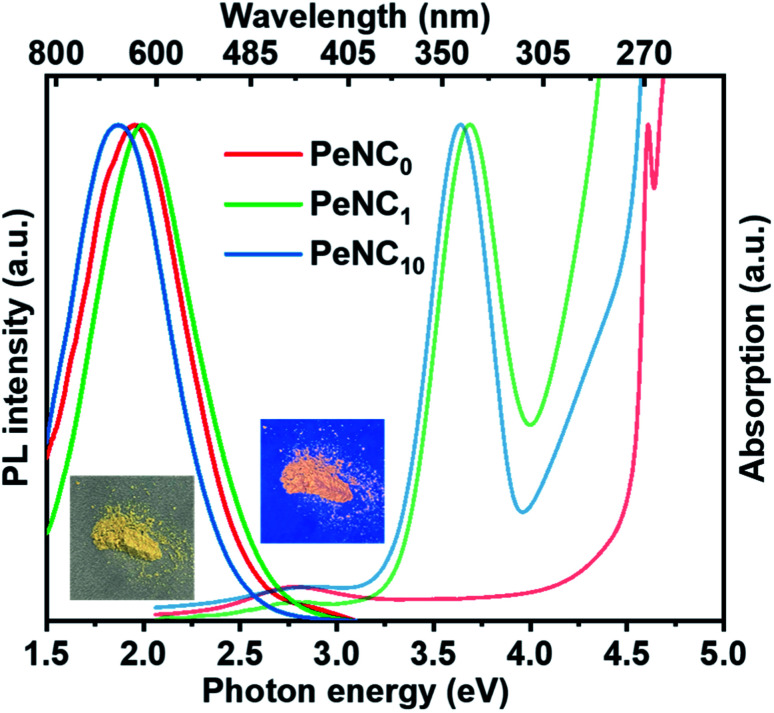
UV-Vis absorption and PL spectra of PeNC_0_ (*λ*_ex_ = 275 nm), PeNC_1_ (*λ*_ex_ = 330 nm), and PeNC_10_ (*λ*_ex_ = 330 nm) with nanocrystals in the powder form. Photographs showing the PeNC_1_ powder sample under room illumination (left) and UV irradiation (right).

The optical properties of Cs_2_Ag_0.65_Na_0.35_InCl_6_ NCs were studied by UV-Vis and PL spectroscopies. Also, the resultant optical absorption and PL spectra of the Cs_2_Ag_0.65_Na_0.35_InCl_6_ NC samples were determined and are shown in Fig. 3. Cation doping has complicated impacts on the perovskite nanocrystals because even a very small substitution of In^3+^ by Bi^3+^ would greatly affect the bandgap and exciton radiation channel. For the undoped sample PeNC_0_, the conduction band minimum (CBM) was mainly contributed by In-5s and Cl-3p, and the valence band maximum (VBM) was contributed by Ag-4d and Cl-3p.^45^ When Bi^3+^ ions were doped into the NCs, it was noted that an additional excitonic absorption peak was presented near 3.7 eV, which was attributed to band-edge absorption where Bi could be the localization center for electrons at the conduction band edges.^55,68^ With the incremental addition of Bi ions from 2% to 15%, the optical bandgap estimated by the Tauc plots (Fig. S4†) narrowed from 3.47 eV to 3.41 eV, which was caused by the lower CBM of the p-orbital derived from Bi cations.^69^ The excitation energy was obtained from the photoluminescence excitation (PLE) peak value (Fig. S5†). The PL spectrum of the PeNC_0_ was observed by excitation with 4.51 eV, while the PL spectra of PeNC_1_ and PeNC_10_ were obtained by excitation at 3.76 eV. The red-shift in the PL spectra of PeNC_10_ compared to PeNC_1_ corresponded to the narrowed bandgap. The highest PLQY of 33.2% was observed for PeNC_1_, while it was 3% for PeNC_0_ and 15.5% for PeNC_10_ as shown in Table 1 and Fig. S6.†

**Table 1:** Decay parameters of the PeNCs at room temperature^a^

Sample	PLQY (%)	*τ*_1_ (μs) (*A*_1_%)	*τ*_2_ (μs) (*A*_2_%)	*τ*_avg_ (μs)	*k*_r_ (μs^−1^)	*k*_nr_ (μs^−1^)	*R*-Square
PeNC_0_	3	0.23	—	0.23	0.13	4.20	0.998
PeNC_1_	33.2	1.10 (79%)	7.26 (21%)	2.40	0.14	0.28	0.973
PeNC_10_	15.5	0.81 (80%)	3.53 (20%)	1.36	0.11	0.62	0.999

a PLQY: photoluminescence quantum yield; *τ*_1_-short and *τ*_2_-long lifetime; *A*_1_ and *A*_2_ are the contributions for *τ*_1_ and *τ*_2_, respectively; *τ*_avg_: average lifetime, which was calculated by *τ*_avg_ = *τ*_1_*A*_1_ + *τ*_2_*A*_2_; *k*_r_-radiative recombination rate and *k*_nr_-nonradiative recombination rate, which were calculated by *k*_r_ = PLQY/*τ*_avg_ and *k*_nr_ = 1/*τ*_avg_ − *k*_r_, respectively.

**Fig. 4:**
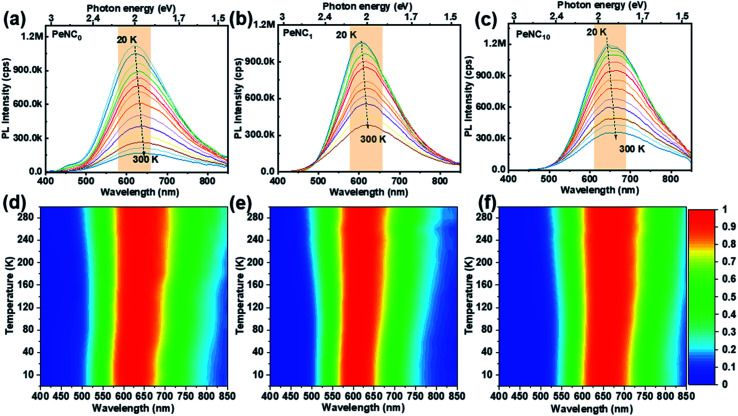
Temperature-dependent PL spectra of (a) PeNC_0_ (*λ*_ex_ = 275 nm), (b) PeNC_1_ (*λ*_ex_ = 330 nm), and (c) PeNC_10_ (*λ*_e*x*_ = 330 nm) from 20 to 300 K. Normalized PL intensity mapped with the emission wavelength and temperature for (d) PeNC_0_ (e) PeNC_1_, and (f) PeNC_10_.

To discuss a possible mechanism of the doping effect on the PL performance, the PL spectra of PeNC_0_, PeNC_1_, and PeNC_10_ in powder form were measured in the temperature range between 20 K to 300 K. Fig. 4 shows the variation in the PL spectra of PeNC_0_, PeNC_1_ and PeNC_10_ photoexcited at 4.51, 3.76, and 3.76 eV, respectively. It is worth mentioning that there was a weak peak at around 2.76 eV for the PeNC_0_ (see Fig. 4a), which could be attributed to the free-exciton emission.^45^Fig. 4a–c shows the decreasing trend in the PL intensity of all the three samples with the temperature rise. Meanwhile, the samples exhibited a red-shift in the PL peak when the temperature was increased from 20 K to 100 K. Interestingly, this behavior is the opposite of most reported perovskite NCs.^64,70–72^ There are a few papers showing a red-shift trend, but those crystals are known to show the temperature-dependent phase transition or halide-type effect.^63,73^ However, the results of the LT-XRD measurements showed that all three samples maintained an ideal cubic perovskite structure and unchanged crystal phase in the temperature range between 5 K and 300 K. As mentioned before, the broad emission of the Cs_2_Ag_0.65_Na_0.35_InCl_6_ NCs originated from STE emission. Herein we propose that the red-shift of the PL peak could be attributed to the narrowing emission bandgap of the self-trapped state with the temperature increasing, which resulted from the lattice dilatation and electron–phonon interaction, similar to conventional semiconductor materials.^74^

**Fig. 5:**
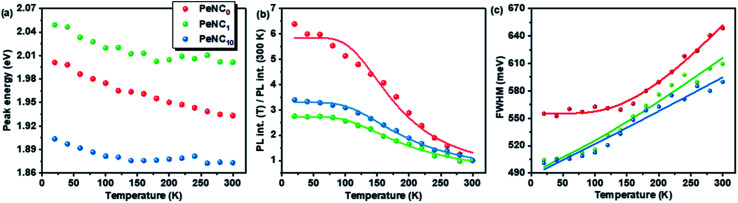
Temperature-dependent PL (a) emission peak energy, (b) peak intensity, and (c) FWHM of PeNC_0_, PeNC_1_, and PeNC_10_.

The variation in the PL peak energy with temperature from 20 K to 300 K for the three samples is shown in Fig. 5a. The PL peak energies of the NC powders for PeNC_0_, PeNC_1_, and PeNC_10_ were determined to be 2.00, 2.05, and 1.90 eV at 20 K and 1.93, 2.00, and 1.87 eV at 300 K, respectively. It was seen that the PL peak energy of PeNC_0_ decreased significantly (0.25 meV K^−1^) while the other two doped samples (*i.e.* PeNC_1_ and PeNC_10_) exhibited smaller shifts of 0.18 and 0.11 meV K^−1^ with the increasing temperature. On the other hand, the integrated PL intensities for the PeNC_0_, PeNC_1_, and PeNC_10_ samples slightly decreased with the increasing temperature as shown in Fig. 5b, yielding PL quenching. The successive decreasing behaviors of the PL intensity with the rise in temperature appeared due to the thermal activation of the nonradiative channels present in the NCs. According to this context, PeNC_0_ contained the largest amounts of defects as nonradiative channels, whereas PeNC_1_ had the smallest defect density (Scheme 1), consistent with the difference in PLQY. The temperature-dependent PL intensities could be fitted well according to the Arrhenius formula:^75^1
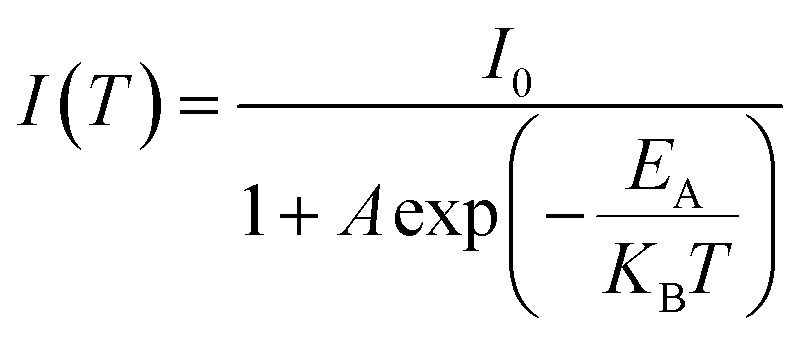
where *I*_0_ is the PL intensity at 20 K, *A* is the proportional constant, *E*_A_ is the activation energy, and *K*_B_ is the Boltzmann constant. The estimated values of thermal activation energy were 55.8, 46.3, and 48.8 meV for the PeNC_0_, PeNC_1_, and PeNC_10_ samples.

It can be seen from Fig. 4d–f that the PL full-width at half maximum (FWHM) was broadened for each sample with the temperature increasing, which was attributed to the strong exciton–phonon coupling. As shown in Fig. 5c, the PL FWHM was fitted by adapting the independent Boson model:^76^2
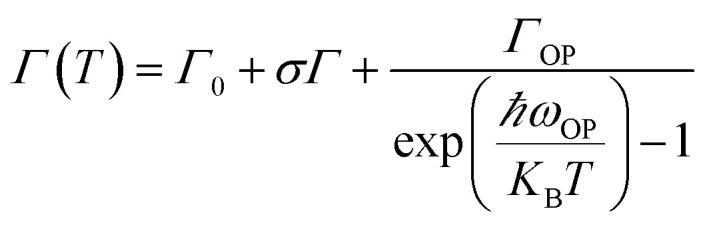
where *Γ*_0_ is the inhomogeneous broadening contribution, *σ* and *Γ*_OP_ describe the interactions of the exciton–acoustic phonon interaction and the exciton–optical phonon interaction contributions to the FWHM broadening, respectively, and *ℏω*_OP_ is the optical phonon energy. The corresponding fitted values are listed in Table 2, where it can be seen that the fitted optical phonon energies of PeNC_0_, PeNC_1_ and PeNC_10_ were 47.9, 41.2, and 42.0 meV, respectively. These values were similar to the bulk phonon energy (∼50 meV).^79^ Smaller optical phonon energy values of the doped samples imply that more phonons are produced, which would serve as scattering centers,^77^ possibly due to the strained crystalline lattice by doped ions. The inhomogeneous broadening of PL FWHM might be contributed by the surface defect-induced trap state.^78^ Also, the inhomogeneous broadening factor of PeNC_0_ (555 meV) was higher than that of PeNC_1_ (490 meV), which indicated that the defect density was suppressed after Bi-doping. Electron–phonon coupling is also crucial to the formation of STEs, where, because of the “soft” lattice nature of the octahedral halide coordination in the double-perovskite structure, strong electron–phonon coupling results in elastic structural distortion, leading to a broadband STE emission.^48^ A highly efficient radiative recombination process thus requires a suitable electron–phonon coupling effect. The electron–phonon coupling could be quantitatively characterized by Huang–Rhys’ factor *S*. The *S* value is normally small in free-exciton materials, such as CdSe^80^ and CsPbBr_3_,^81^ but larger than 10 in STE materials. As shown in Fig. S7,† the fitted *S* values for PeNC_0_, PeNC_1_, and PeNC_10_ were 24.1, 27.7, and 26.7, respectively, which were smaller than bulk crystals (∼40)^45^ but larger than reported NCs (15.5).^54^ It was demonstrated that Cs_2_Ag_0.65_Na_0.35_InCl_6_ NCs possessed enough electron–phonon coupling, which is beneficial for the formation of STE emission, to achieve a high PLQY.

**Table 2:** Physical parameters obtained from fitting the experimental temperature-dependent PL data^a^

Sample	*Γ*_o_ (meV)	*σ* (meV K^−1^)	*E*_ph_ (meV)	*Γ*_op_ (meV)	*E*_A_ (meV)
PeNC_0_	555.4 ± 2.2	1.33 × 10^−13^	47.9	519.3 ± 22.0	55.8 ± 6.2
PeNC_1_	489.7 ± 7.1	0.353 ± 0.069	41.2	80.3 ± 70.7	46.3 ± 2.3
PeNC_10_	487.1 ± 6.0	0.345 ± 0.068	42.0	16.7 ± 72.0	48.8 ± 2.4

a *Γ*_o_: inhomogeneous broadening; *σ*: coupling coefficient of exciton–acoustic phonons; *E*_ph_: longitudinal optical (LO) phonon energy; *Γ*_op_: coupling strength of exciton–LO phonons; *E*_A_: activation energy.

**Fig. 6:**
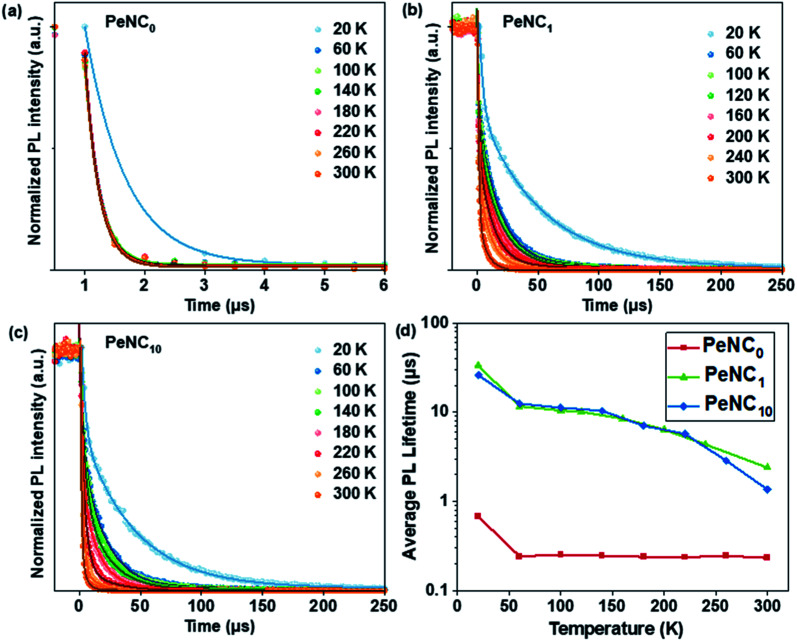
TR-PL curves of (a) PeNC_0_ (b) PeNC_1_, (c) PeNC_10_ from 20 to 300 K and (d) their average lifetimes extracted from biexponential fitting at various temperatures.

Besides the broadband and large Stokes shift emission, a long radiative PL lifetime is also a specific property of STEs, and is shown in Fig. 6a–c. To study the effect of doped Bi on the charge-carrier lifetime as a function of temperature, we measured time-resolved PL as a function of temperature for the PeNC_0_, PeNC_1_, and PeNC_10_ samples between 20 K and 300 K. As shown in Fig. 6d, the average lifetimes of PeNC_1_ and PeNC_10_ were longer than that of PeNC_0_ at all the temperatures, while the short lifetime component was ascribed to the trap-assisted nonradiative recombination channel, which when combined with the calculated nonradiative recombination rate in Table 1 allows concluding that the Bi-doping passivates the defects and suppresses the nonradiative recombination rate. The temperature-dependent average lifetimes of exciton decay for the Bi-doped samples were calculated by fitting the data to a biexponential fit (Table S2†) with a long-lived component (3–50 μs) and a less-long-lived component (0.8–3 μs), while the PL decay of undoped NCs was fitted by a mono-exponential function with a less-long-lived component (0.23 μs). The R-square values of the three plots were larger than 0.97, indicating a good fit. As shown in Fig. 6d, all three samples demonstrated that the changes in the lifetime were shortened a lot from 20 K to 60 K, but quite mild in the range from 60 K to 300 K, which could be described as the Boltzmann distribution,^82^ in accordance with the reported broadband-emission double perovskites.^52^ The proportion of the less-long-lived components *versus* temperature is shown in Fig. S8,† and this contribution became prominent when the temperature increased in both PeNC_1_ and PeNC_10_. Combined with the decreased PL intensity when the temperature increased, the less-long-lived component could be ascribed to fast nonradiative losses, likely due to the defects, while the long-lived part was contributed by the recombination of the STE. This phenomenon has also been observed in other double-perovskite nanocrystals.^53^ As a result, the average PL lifetimes decreased with the increasing temperature due to the increasing contribution of the faster nonradiative decay channels.

## 4. Conclusions

We synthesized undoped and bismuth-doped Cs_2_Ag_0.65_Na_0.35_InCl_6_ double-perovskite NCs to investigate the role of the dopant on the enhanced PL performance through LT-XRD and low-temperature PL spectroscopic studies. The LT-XRD characterization indicated the successful doping and showed that there was no phase transition between 5 K and 300 K. The absolute value of PLQY was as high as 33.2%, which was obtained by 2% bismuth doping. The temperature-dependent PL study suggested that the diminished nonradiative channels brought about the increase in the PLQY. The PL linewidth broadening from 20 K to 300 K of the NCs was explained by electron–phonon coupling.

## Funding sources

JSPS KAKENHI Grant-in-Aid for Scientific Research (B) Grant Number 21H01910 and 21H01743, The Murata Science Foundation. JSPS KAKENHI (Grant Number 21K18942).

## Author contributions

X. H. performed research; Y. M. measured the samples with XRD at different temperatures; all the authors discussed the results, and X. H. and N. S. wrote the paper.

## Conflicts of interest

There are no conflicts to declare.

## Supplementary Material

NA-004-D2NA00238H-s001
