# Supplementary material for: Impact of bismuth-doping on enhanced radiative recombination in lead-free double-perovskite nanocrystals
Source: Nanoscale Adv. 2022 Jun 24;4(14):3091–100. doi: 10.1039/d2na00238h (PMC9419852; doi:10.1039/d2na00238h)
Supplement: NA-004-D2NA00238H-s001 [file NA-004-D2NA00238H-s001.pdf]

Electronic Supplementary Material (ESI) for Nanoscale Advances.
This journal is © The Royal Society of Chemistry 2022

Impact of Bismuth-Doping on the Enhanced Radiative Recombination in Lead-free Double Perovskite Nanocrystals

Author name: *Xiaoyu Huang*[1,2]*, Yoshitaka Matsushita*[3]*, Hong-Tao Sun,*[1] *Naoto Shirahata*[1,2,4]*

Author address:

[1]International Center for Materials Nanoarchitectonics (MANA), National Institute for Materials Science (NIMS), Ibaraki 305-0044, Japan

[2]Graduate School of Chemical Sciences and Engineering, Hokkaido University, Sapporo 060-0814, Japan; Department of Physics, Chuo University, Tokyo 112-8551, Japan

[3]Research Network and Facility Services Division, National Institute for Materials Science (NIMS), Ibaraki 305-0047, Japan

[4]Department of Physics, Chuo University, Tokyo 112-8551, Japan

Email: SHIRAHATA.Naoto@nims.go.jp

KEYWORDS: Lead-free double perovskite, nanocrystal, bismuth doping, photoluminescence

Supporting Figure

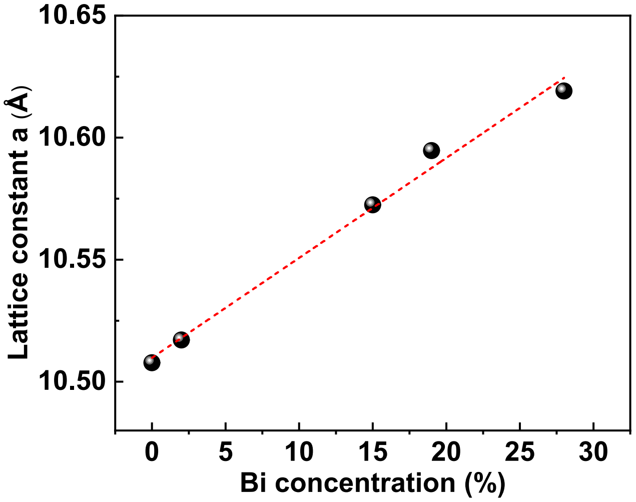

Figure S1. The lattice constant as function of Bi concentration in $Cs_2Ag_{0.65}Na_{0.35}InCl_6$ NCs. (PLQY: 11.5% for 19% Bi-doped NCs, and 9.3% for 28% Bi-doped NCs.)

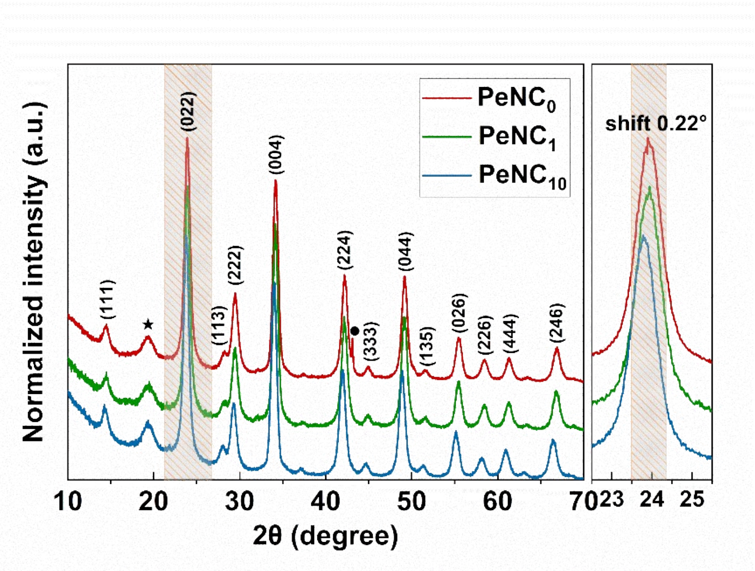

Figure S2. XRD patterns of $PeNC_0$, $PeNC_1$ and $PeNC_{10}$ measured at 5 K. The peaks indicating as ★ and ● are from the X-ray window materials of the cryostat and from Cu sample holder, respectively.

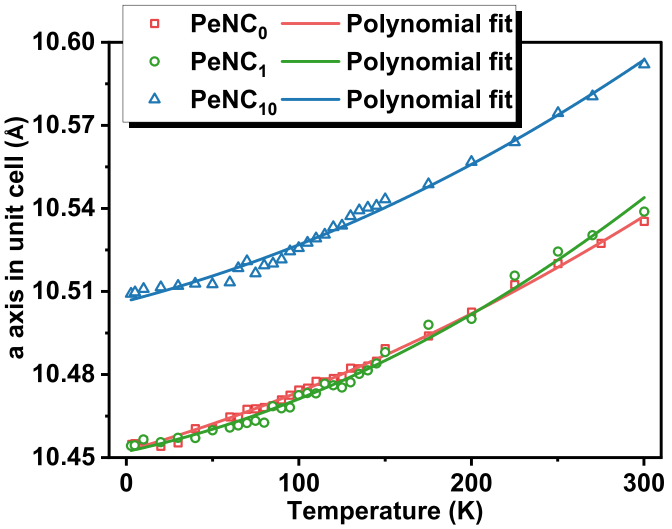

Figure S3. Thermal expansion coefficient fitting of $PeNC_0$, $PeNC_1$ and $PeNC_{10}$.

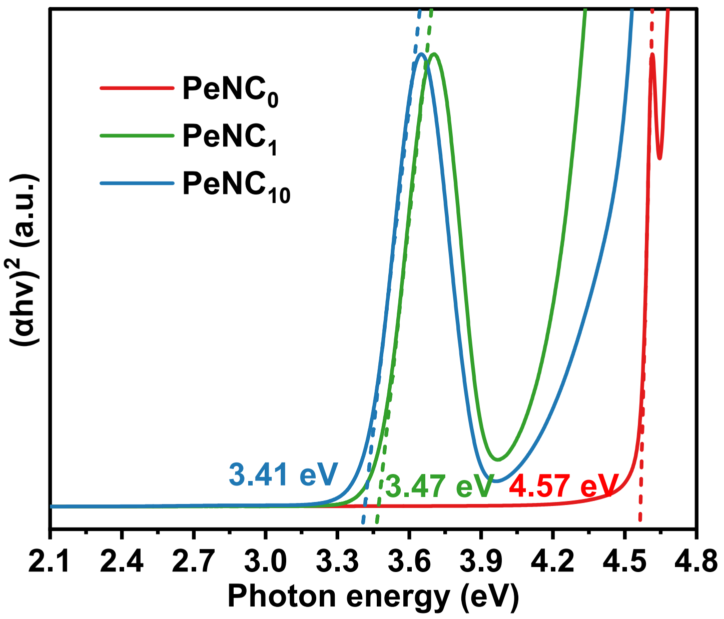

Figure S4. $(\alpha h\nu)^2$- hν plots of $PeNC_0$ (red), $PeNC_1$ (green) and $PeNC_{10}$ (blue). The values of Eg extracted from extrapolations to $\alpha = 0$.

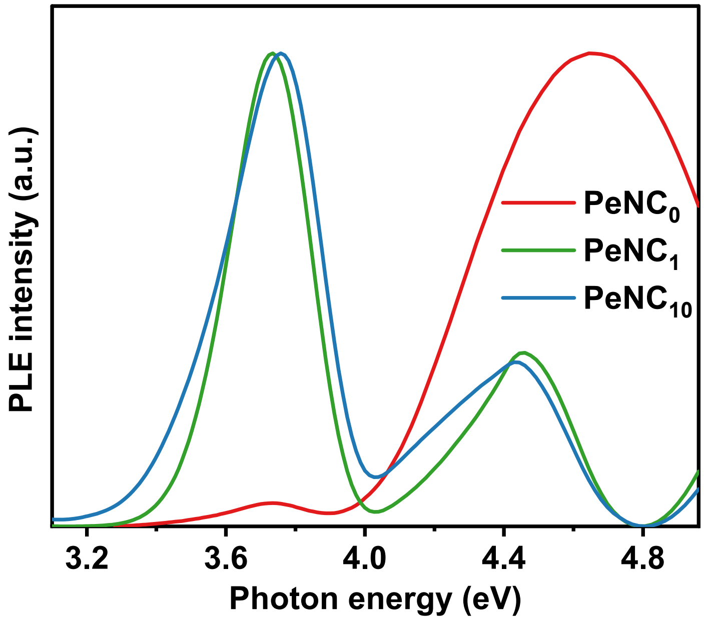

Figure S5. Excitation spectra of photoluminescence measured for $PeNC_0$ (634 nm-emission peak), $PeNC_1$ (592 nm-emission peak) and $PeNC_{10}$ (630 nm-emission peak).

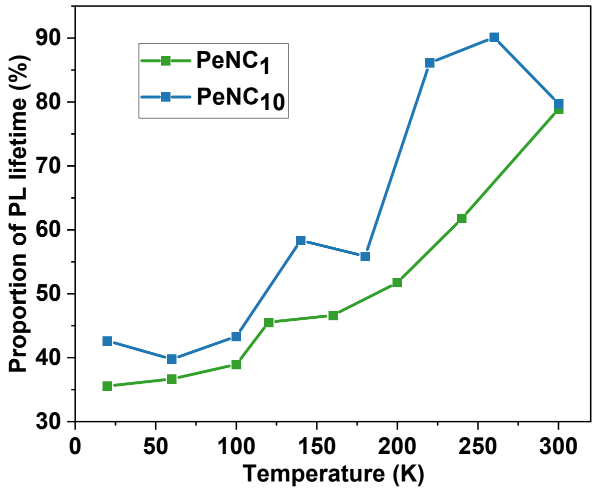

Figure S6. The proportion of less-long lived PL lifetime for $PeNC_1$ (green) and $PeNC_{10}$ (blue) dependent on temperature.

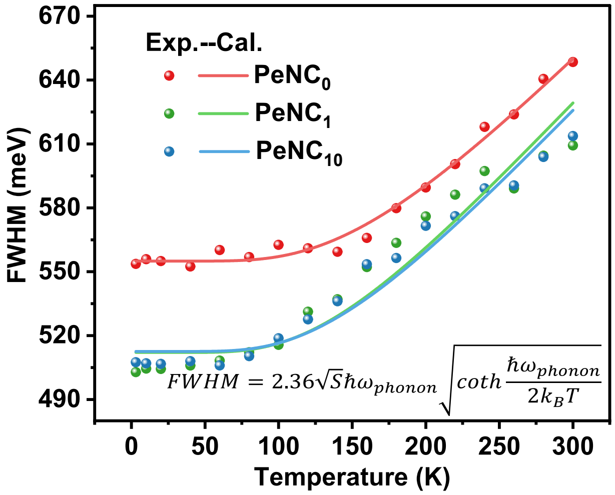

Figure S7. Temperature-dependent PL FWHM of $PeNC_0$, $PeNC_1$ and $PeNC_{10}$.

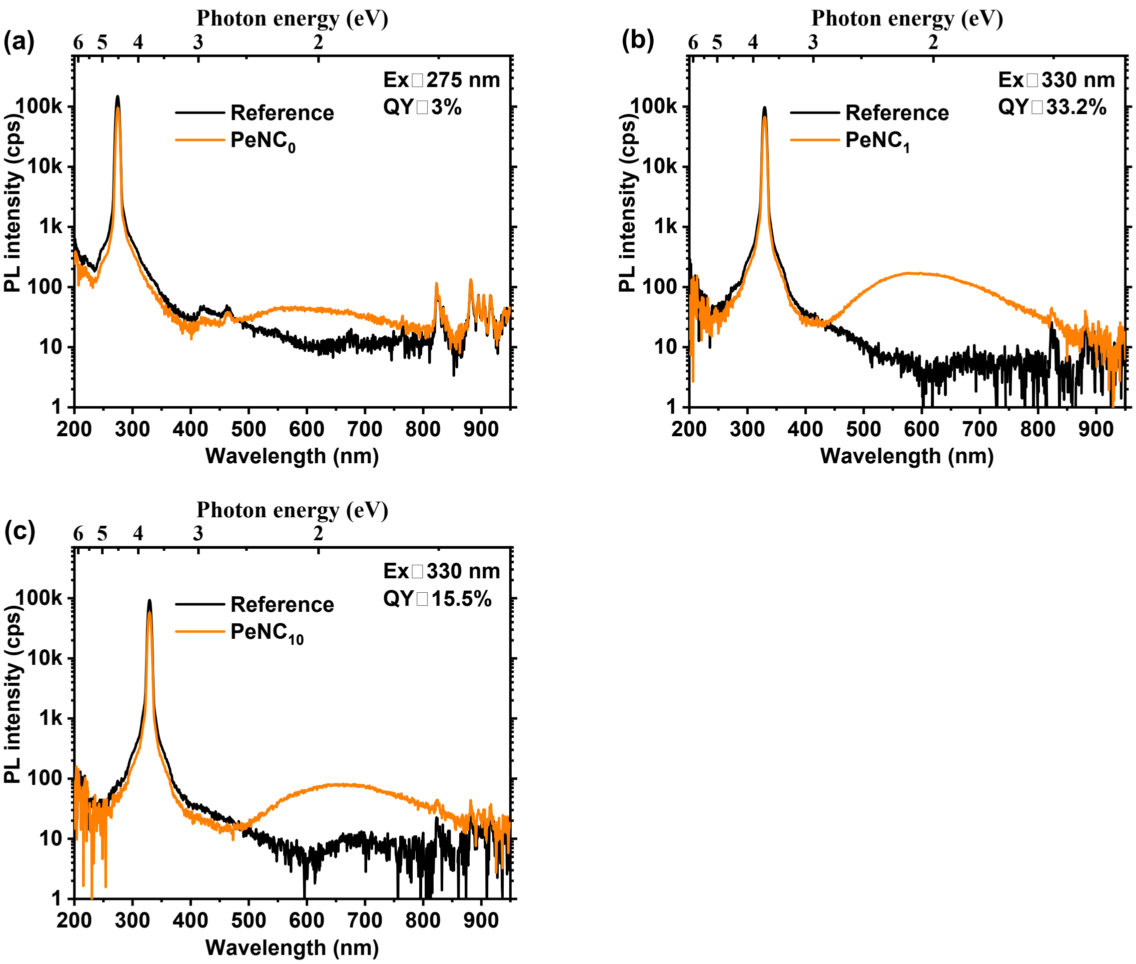

Figure S8. PLQY for $PeNC_0$ (a), $PeNC_1$ (b) and $PeNC_{10}$ (c).

Supporting Table

Table S1. STEM-EDS elemental analysis on Bi-doped and undoped NCs.

EDS/atom ratio	Cs/In	Ag/(Ag+Na)	Na/(Ag+Na)	In/(In+Bi)	Bi/(In+Bi)	Cl/In
$PeNC_0$	2.32	0.66	0.35	1	0	5.61
$PeNC_1$	2.39	0.65	0.35	0.98	0.02	5.50
$PeNC_{10}$	2.48	0.63	0.37	0.85	0.15	4.86

Table S2. Fitted decay parameters of PeNCs at different temperature.

Temperature (K)		20	60	100	140	180	220	260	300
$PeNC_1$	τ_1 (μs) (A_1%)	3.13 (36%)	0.97 (37%)	1.06 (39%)	1.65 (46%) @ 120 K	0.95 (47%) @ 160 K	1.00 (52%) @ 200 K	1.08 (62%) @ 240 K	1.10 (79%)
	τ_2 (μs) (A_2%)	50.56 (64%)	17.82 (63%)	16.48 (61%)	17.20 (54%) @ 120 K	14.99 (53%) @ 160 K	12.14 (48%) @ 200 K	9.65 (38%) @ 240 K	7.26 (21%)
	R-square	0.998	0.996	0.996	0.991	0.994	0.992	0.985	0.971
$PeNC_{10}$	τ_1 (μs) (A_1%)	2.99 (43%)	2.19 (40%)	2.24 (43%)	3.87 (58%)	1.92 (56%)	3.45 (86%)	1.82 (90%)	0.81 (80%)
	τ_2 (μs) (A_2%)	43.52 (57%)	19.27 (60%)	18.16 (57%)	19.52 (42%)	13.51 (44%)	19.70 (14%)	12.50 (10%)	3.53 (20%)
	R-square	0.999	0.998	0.997	0.993	0.994	0.975	0.978	0.999
